# Machine Learning to Improve the Sensing of Biomolecules by Conical Track-Etched Nanopore

**DOI:** 10.3390/bios10100140

**Published:** 2020-10-05

**Authors:** Nathan Meyer, Jean-Marc Janot, Mathilde Lepoitevin, Michaël Smietana, Jean-Jacques Vasseur, Joan Torrent, Sébastien Balme

**Affiliations:** 1 Institut Européen des Membranes, UMR5635, UM, ENSCM, CNRS, 34095 Montpellier, France; nathan.meyer@umontpellier.fr (N.M.); jmjanot@univ-montp2.fr (J.-M.J.); 2 Mécanismes Moléculaires dans les Démences Neurodégénératives, U1198, UM, EPHE, INSERM, 34095 Montpellier, France; joan.torrent@inserm.fr; 3 Institut des Matériaux Poreux de Paris UMR8004, CNRS, ENS, ESPCI, 75005 Paris, France; mathilde.lepoitevin@ens.fr; 4 Institut des Biomolécules Max Mousseron, Université de Montpellier, CNRS, ENSCM, 34095 Montpellier, France; michael.smietana@umontpellier.fr (M.S.); jean-jacques.vasseur@umontpellier.fr (J.-J.V.)

**Keywords:** nanopore, machine learning, DNA sensing

## Abstract

Single nanopore is a powerful platform to detect, discriminate and identify biomacromolecules. Among the different devices, the conical nanopores obtained by the track-etched technique on a polymer film are stable and easy to functionalize. However, these advantages are hampered by their high aspect ratio that avoids the discrimination of similar samples. Using machine learning, we demonstrate an improved resolution so that it can identify short single- and double-stranded DNA (10- and 40-mers). We have characterized each current blockade event by the relative intensity, dwell time, surface area and both the right and left slope. We show an overlap of the relative current blockade amplitudes and dwell time distributions that prevents their identification. We define the different parameters that characterize the events as features and the type of DNA sample as the target. By applying support-vector machines to discriminate each sample, we show accuracy between 50% and 72% by using two features that distinctly classify the data points. Finally, we achieved an increased accuracy (up to 82%) when five features were implemented.

## 1. Introduction

For the past three decades, single nanopore technology have emerged as single-molecule sensors and offer many practical uses such as long read DNA sequencing [1,2]. This was achieved by engineering biological nanopores combined with biological machines to control the DNA translocation speed [3,4,5,6,7]. Beside sequencing, biological nanopores provide a nice platform to analyze the DNA substructure such as hairpin [8], the hybridization [9,10], zipping [11] or the interaction with protein [12]. At the beginning of the 2000s, the idea to mimic biological nanopores was demonstrated using different types of thin film. First, thin films of semiconductors (SiN) drilled by transmission electron microscopy or focused ion beam were used to provide nanopores with a low aspect ratio [13,14]. Next, polymer nanopore obtained by the track-etched technique provided a long high-aspect-ratio nanochannel [15,16]. More recently, 2D materials with reduced thickness down to a couple of angstroms, such as metal nitride or oxide, were developed to improve the noise and/or wettability of those low-aspect-ratio nanopore [17,18].

Regardless the type of artificial nanopore (solid-state or polymer), their performance in terms of precision is lower than the biological ones [19]. However, they offer pores of various sizes from a couple of nm up to hundreds of nm allowing the detection of folded proteins, protein assemblies and nanoparticles [20,21,22]. Artificial nanopores can be classified according to their aspect ratio. The 2D materials are the most promising to discriminate single nucleotides with potential applications in DNA sequencing [23,24]. The SiN and other nitride-based materials are the most used for single molecule sensing. They can be drilled by dielectric breakdown making this approach a low-cost technology [25,26]. Their aspect ratio allows discriminating the length of DNA as well as protein shape based on the amplitude of the current blockade but also the dwell time [27,28,29,30,31,32,33]. Solid-state nanopores also show interesting applications in the characterization of DNA knots and DNA-protein binding [34,35,36]. The track-etched nanopores have low resolution but their µm length scale allows increasing the dwell time making the polymer detection easier [37,38]. Even if track-etched nanopores are much less used than SiN membrane nanopores, several biomacromolecules were successfully detected such as proteins [37], DNA from 10 to 100 bp [39,40,41,42], hyaluronic acid [21] and amyloid [33,43]. In addition, they are mechanically robust, with expanded lifetimes up to several weeks [33]. This is particularly useful to investigate the kinetic of protein aggregation as well as their enzymatic degradation [20]. Furthermore, since the dwell time is enhanced in the track-etched nanopore [38], transient conductivity events can be easily detected without the use of a MHz amplifier. Nevertheless, their main limitation is their low resolution to discriminate small polymers. In order to improve their performance, their surfaces can be easily functionalized to tune their properties. Among them, the partial conversion of carboxylic acids into amine moieties [44], the deposition of Al_2_O_3_ to tune the pore size [40] or the direct insertion of a biological nanopore [38]. Despite interesting results, the question of how the resolution of track-etched nanopore can be improved without chemical functionalization is still open. By tackling the problem of low resolution, the track-etched nanopore could offer a powerful platform to analyze the DNA size and structure. Indeed, compared to biological nanopore the tip diameter can be tuned to be sensitive to double-strand DNA or chain structure (i.e., knot, or hairpin). In this case, we could consider to determinate the ratio of different structures. Another advantage is the facility to modify the nanopore entrance to generate a specific interaction.

A way to increase resolution of nanopores is the use of machine learning algorithms [45,46], such as those used for DNA sequencing [47,48,49,50]. Moreover, combining the high resolution of biological nanopores and machine learning is a powerful tool to improve the nanopore resolution [51,52] allowing the identification of the protein domains [53], DNA base modification [54] and the C5 cytosine variant of DNA [55]. Here, we sought to apply classic algorithms of machine learning in the case of track-etched nanopores. Usually, the molecule detection by nanopore is characterized by two parameters: the relative current blockade and dwell time. The area of the events is also sometimes considered. There is compelling evidence using SiN nanopores that all these parameters are more or less correlated. We addressed the hypothesis that machine learning, in conjunction with the careful choice of multiple parameters that allow for characterizing the current blockade events, could improve the accuracy of DNA discrimination.

Here, we aim to demonstrate that classic algorithms of machine learning are powerful methods to data analysis of the nanopore sensing experiment. To do so, we have selected oligonucleotides with well-defined DNA sequences (A_40_/T_40_, T_40_ and A_10_/T_10_) as small macromolecules. Their detection was achieved through non-functionalized conical nanopore obtained by the track-etched method. The latter has a low resolution to discriminate small macromolecules. It is thus an ideal candidate to evaluate the benefits of the machine learning approach. From the parameter of the current blockade, we establish the correlation degree and then evaluate the accuracy of nanopores to discriminate the sample.

## 2. Material and Methods

### 2.1. Material

The A_10_, A_40_, T_10_ and T_40_ were obtained as previously reported [40]. Briefly, they were synthesized from commercially available phosphoramidite building blocks (Link Technologies Ltd., Bellshill, Scotland) in a 1 μmol scale using an ABI 381A DNA synthesizer by standard phosphoramidite chemistry. Then they were purified by RP-HPLC and characterized by MALDI-TOF MS.

### 2.2. Track-Etched Nanopore Design

Single conical nanopore was obtained by the track-etched method under dissymmetrical condition as previously reported [56]. Briefly, the single tracks were produced by Xe irradiation (8.98 MeV u-1) (GANIL, SME line, Caen, France) of polyethylene terephthalate (PET) film (thickness 13 µm, biaxial orientation ES30_10_61 Goodfellow). The tracks were activated by UV exposition 12 h per side (Fisher bioblock; VL215.MC, λ = 312 nm) before chemical etching process. The etching of conical nanopore was performed under dissymmetric condition (etchant solution 9 M NaOH and stop solution 1 M KCl 1 M of acetic acid) using the electrostopping method (1 V). After nanopore opening, the tip diameter (*d_t_*) of conical nanopores was calculated from the dependence of the conductance *G* (measured from −100 mV to 100 mV) with KCl concentration 1M, assuming bulk-like ionic conductivity inside the nanopores using Equation (1).

(1) $$G=\frac{\kappa \pi d_{t}d_{b}}{4L}$$

where κ is the conductivity of the solution, L the nanopore length (13 µm) and *d_b_* the diameter of the base side. *d_b_* is calculated from the total etching time *t* using the relationship $d_{b}=2.5t$. The factor 2.5 was determined in our laboratory using multipore membrane track. The pore dimensions used here are $d_{t}=3\;\mathrm{nm}$ $d_{b}=200\;\mathrm{nm}$ α = 0.4° (noted pore 1) and $d_{t}=4\;\mathrm{nm}$ $d_{b}=350\;\mathrm{nm}$ α = 0.8° (noted pore 2).

### 2.3. DNA Detection and Analysis

The DNA strands were detected using resistive pulse methods [57,58,59]. Briefly, the single conical nanopore was mounted between two Teflon chambers containing the same electrolyte solution (NaCl 3 M, EDTA 1 mM, PBS 50 mM, pH 7.2 or KCl 2 M, EDTA 1 mM, PBS 50 mM, pH 7.2). The current was measured by Ag/AgCl, 1 M KCl electrodes connected to the cell chambers by agar–agar bridges. The working electrode and ground electrode were located in the trans-chamber (base side of the nanopore) and in the cis chamber (tip side of the nanopore), respectively. Electrical measurement was performed using a patch-clamp amplifier (EPC10 HEKA electronics, Lambrecht, Germany).

The polynucleotide samples were added on the cis chamber (tip side of nanopore) to reach a final concentration of 10 nM. Positive bias (250 mV or 500 mV) was then applied to the trans-chamber. Ion current was recorded at a sampling frequency of 100 kHz (for T_40_ and A_40_/T_40_) or 200 kHz (for A_10_/T_10_). A Bessel filter at 10 kHz is used. Those experiments were repeated at least 10 times in 8 successive days for each nanopore. The data analysis was performed using a custom-made LabView software with Butterworth filter of 2.5 kHz, 2 orders. The base line fluctuation was corrected using a Savitzky–Golay filter of 2400 side points, 1 order. The detection event was performed using a threshold of 3σ (σ where is the standard deviation of the signal). Each event was characterized by the relative current blockade (Δ*I/I*_0_), the dwell time (Δ*t*), the area (*AUC*), the right (*RS*) and left slopes (*LS*). The parameters of the current blockade were analyzed using Matlab and the toolbox “statistical and learning machine”.

## 3. Results and Discussion

**Figure 1:**
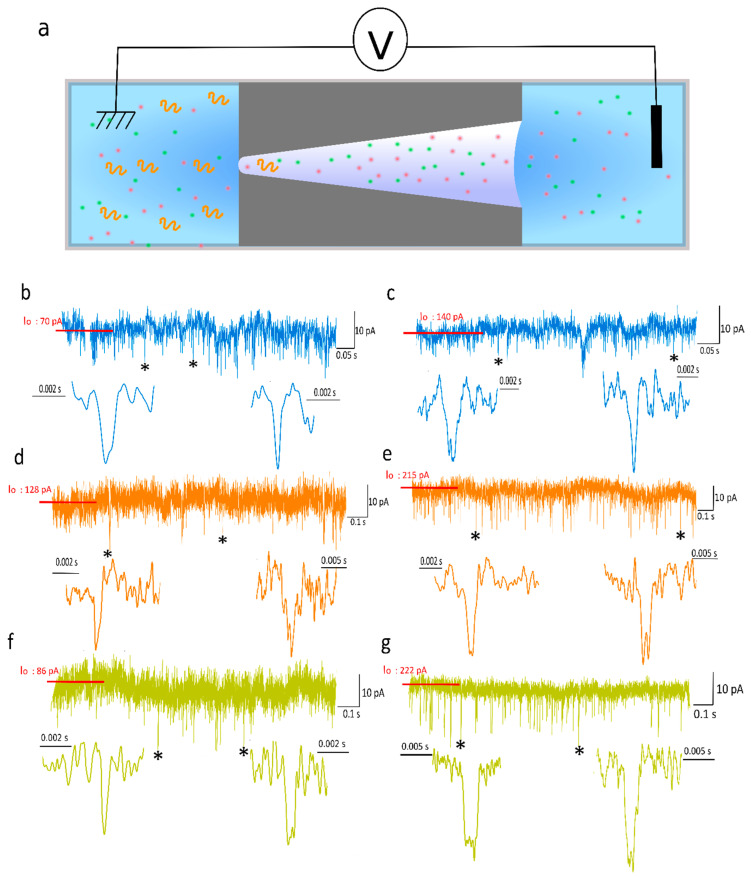
(**a**) Sketch of DNA sensing using conical nanopore. Examples of current traces and zooms of current blockade recorded for A_10_/T_10_ at (**b**) 250 mV and (**c**) 500 mV, A_40_/T_40_ at (**d**) 250 mV and (**e**) 500 mV, T_40_ at (**f**) 250 mV and (**g**) 500 mV. The symbol * identifies the examples of current blockade zoom below each trace. The current traces were obtained using pore 1 *d_t_* = 3 nm, *d_b_* = 200 nm.

The experimental detection of all DNA samples A_10_/T_10_, A_40_/T_40_ and T_40_ were performed from the tip side to the base side under two different electrolyte conditions (NaCl 3 M, EDTA 1 mM, PBS 50 mM, pH 7.2 or KCl 2 M, EDTA 1 mM, PBS 50 mM, pH 7.2) (Figure 1a). Figure 1b–g shows examples of current traces recorded at 250 mV and 500 mV for all samples. From the current traces, the events related to the DNA translocation through the nanopore were detected. These current blockades were usually described by the relative current blockade (Δ*I/I*_0_), which is the ratio between the amplitude of the current blockade and the base line current, and the dwell time. These two parameters were first extracted to characterize all the events recorded during our experiments.

**Figure 2:**
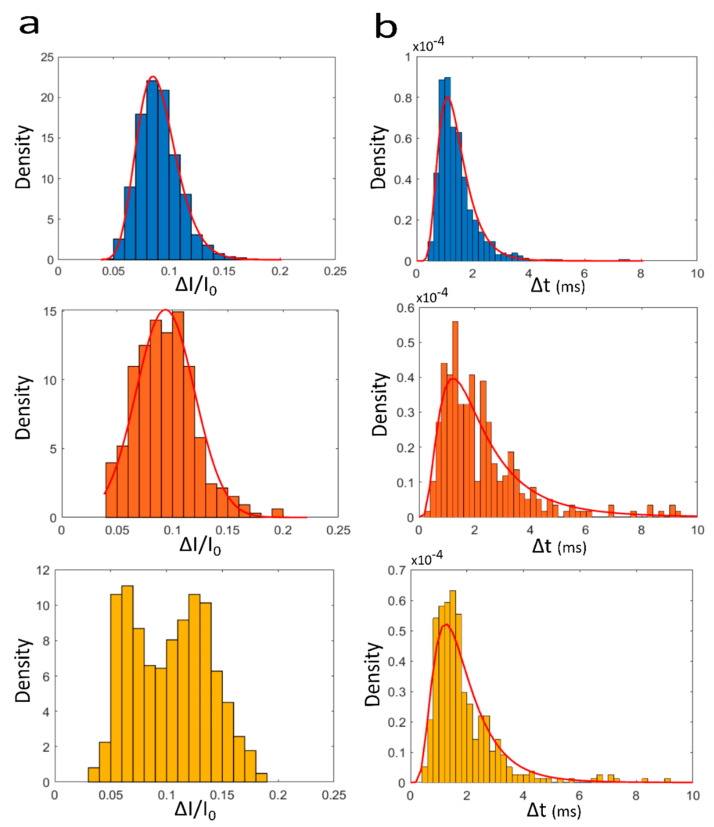
Distribution histograms of (**a**) the amplitude of the relative current blockade (Δ*I/I*_0_) and (**b**) the dwell time (Δ*t*) for the A_10_/T_10_ (blue), A_40_/T_40_: (orange), T_40_ (yellow). The events were recorded at 500 mV, the number of events n = 793, 332 and 634 for A_10_/T_10_, A_40_/T_40_ and T_40_, respectively. The results were obtained using pore 1 *d_t_* = 3 nm, *d_b_* = 200 nm. The density (*d_i_*) is the frequency (*f_i_*) of event relative to the sample size (*n*) and the bin width (w_i_) di=finwi where *w_i_* = 0.01 and 200 for Δ*I/I*_0_ and Δ*t*, respectively.

In Figure 2 are reported the distribution histograms of Δ*I/I*_0_ obtained for the A_10_/T_10_, A_40_/T_40_ and T_40_ at a voltage of 500 mV using pore 1 under KCl 2 M. These distributions are centered at similar values for the three samples: 0.085, 0.093 and 0.066 (another center of distribution is observed at 0.128) for A_10_/T_10_, A_40_/T_40_ and T_40_, respectively. The Δ*I/I*_0_ distributions of the current blockade recorded at 250 mV are centered on 0.15, 0.09 and 0.12 for A_10_/T_10_, A_40_/T_40_ and T_40_, respectively (Figure S1). These values slightly increase for pore 2 (under NaCl 3 M) recorded at 250 mV. The distributions are centered on 0.27, 0.25 and 0.18 for A_10_/T_10_, A_40_/T_40_ and T_40_, respectively (Figure S2). Similar observation can be made for the experiments performed at 500 mV where the centers of distribution are 0.31, 0.28, and 0.36 for A_10_/T_10_, A_40_/T_40_ and T_40_, respectively (Figure S3).

The distribution of Δ*t* for the three samples at 500 mV recorded for pore 1 (and 2) are reported in Figure 2b and Figure S2b. We observe that the distributions are centered close to the same value: 1.07 ms (1.48 ms), 0.95 ms (1.24 ms), and 1.29 ms (1.23 ms) for A_10_/T_10_, A_40_/T_40_ and T_40_, respectively. We notice that the time scale (about 1 ms) is in the same range as the one reported for DNA 50 bp [41]. Under 250 mV, the Δ*t* values do not significantly decrease (Figures S2b and S4b). Indeed, the centers of distribution for pore 1 (and 2) are found to be 1.07 ms (1.39 ms), 0.82 ms (1.25 ms) and 0.87 ms (1.30 ms) for A_10_/T_10_, A_40_/T_40_ and T_40_.

**Figure 3:**
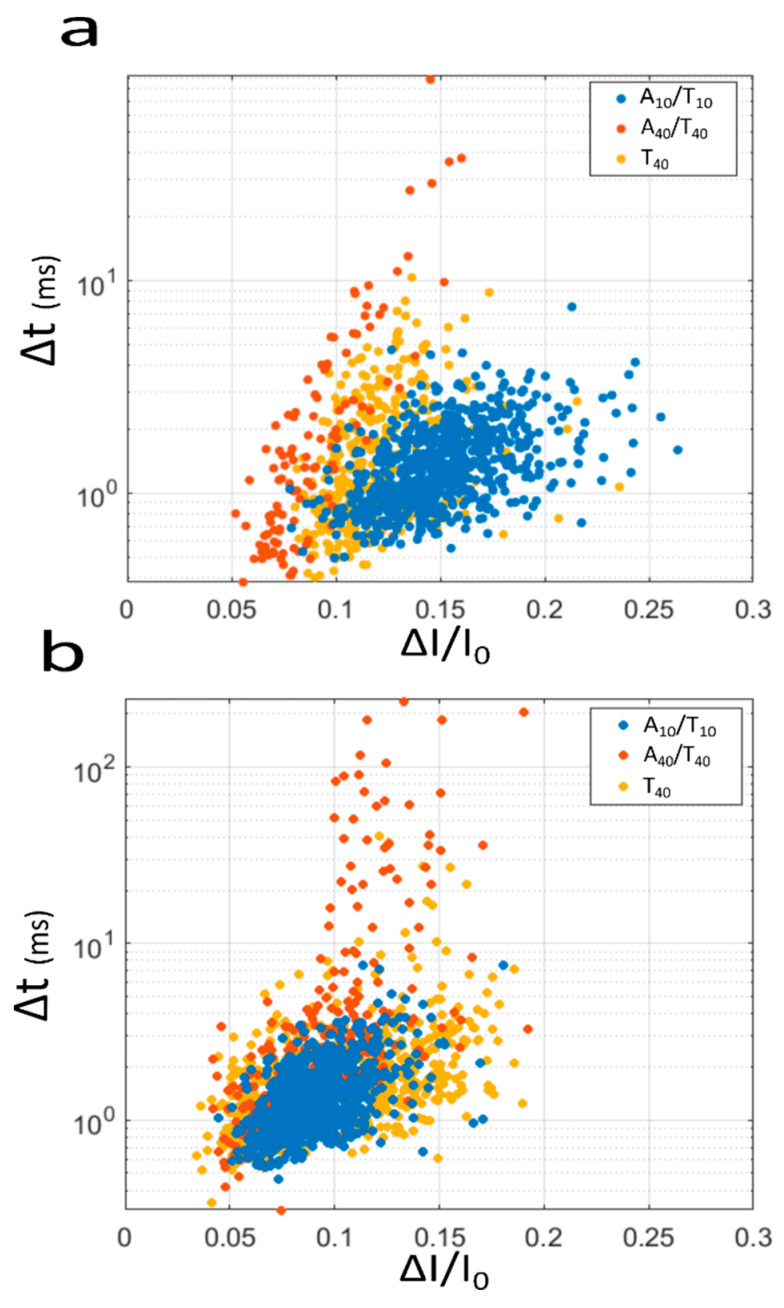
Scatter plot representing the Δ*t* versus the *I/I*_0_ for the A_10_/T_10_ (blue), A_40_/T_40_ (orange), T_40_ (yellow) for a voltage of (**a**) 250 mV and (**b**) 500 mV. The results were obtained using the pore *d_t_* = 3 nm, *d_b_* = 200 nm. The number of events recorded at 250 mV n = 703, 116 and 382 and at 500 mV n = 793, 332 and 640 for A_10_/T_10_, A_40_/T_40_ and T_40_, respectively.

Usually, the Δ*I/I*_0_ and Δ*t* are the main parameters to discriminate the sample analyzed by nanopore sensors. Here, we observe a large overlap between the different distributions (Figure 2 and Figures S1–S3) preventing the sample discrimination. The results also indicate that there is no preferential voltage or pore to discriminate them with only one parameter. In Figure 3 are reported two event maps representing the Δ*I/I*_0_ vs. Δ*t* of translocation events for the three samples at two different voltages: 250 mV (Figure 3a) and 500 mV (Figure 3b) for pore 1. We observe that the cloud of events overlaps due to the similar distribution of Δ*I/I*_0_ and Δ*t*. This overlap makes impossible the discrimination of the DNA samples by a simple clustering analysis. The same trend is observed for pore 2 for the two same voltages (250 and 500 mV, see Figure S4). These observations are not surprising due to the low resolution of track-etched nanopore.

**Figure 4:**
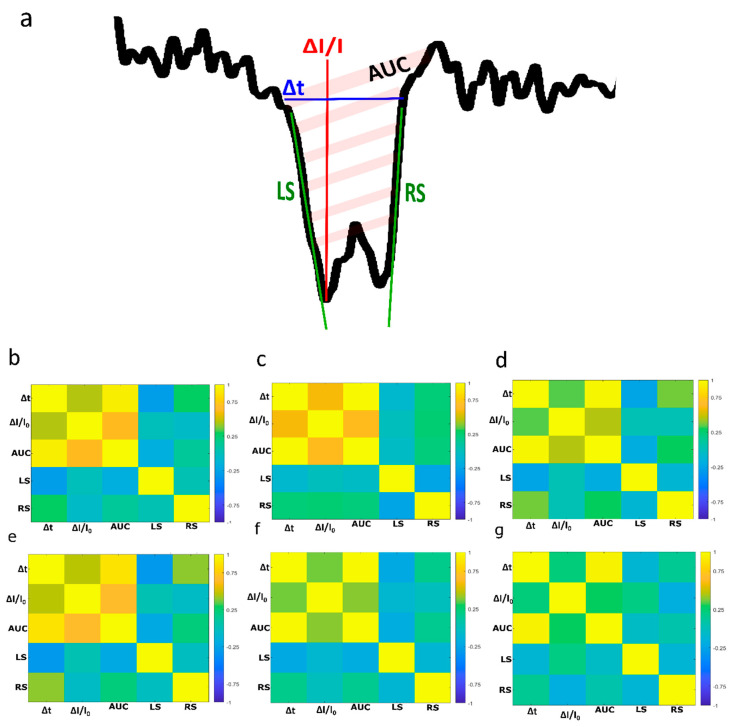
(**a**) Zoom of a translocation event and the representation of variables characterizing events. (**b**–**g**) Heat map representing the correlation between the variables characterizing events obtained under 250 mV for (**b**) A_10_/T_10_, (**c**) A_40_/T_40_, (**d**) T_40_ and at 500 mV for (**e**) A_10_/T_10_, (**f**) A_40_/T_40_, (**g**) T_40_. The results were obtained using the pore *d_t_* = 3 nm, *d_b_* = 200 nm.

To go further, we attempted to define each current blockade with additional parameters (Figure 4a). First, we considered the surface area of the event (AUC) because it takes into account the eventual current fluctuation during the DNA translocation. We could expect that this parameter is strongly correlated to the Δ*t* and the Δ*I/I*_0_. The conical shape of the nanopore can generate a dissymmetrical shape of current blockade events. In that case, the event’s right and left slopes (noted RS and LS, respectively) are expected to be different as previously reported in the case of spherical object [60,61]. Now, we evaluate the correlation degree of these five parameters (Δ*t*, Δ*I/I*_0_, AUC, RS and LR). Usually, a positive correlation between the Δ*t* and the Δ*I/I*_0_ can be observed if the length of the pore is close to that of the analyte. Indeed, this correlation has been reported for protein detection using SiN nanopore [62]. Conversely, in the case of long DNA strands, the amplitudes of the relative current blockade are not correlated with the dwell time since the nanopore is filled with the polymer strand [63]. Here, we report the correlation heat maps of various parameters for the three samples at a voltage of 250 mV (top line) and 500 mV (bottom line) for pore 1 (Figure 4b) and pore 2 (Figure S5). For all samples and regardless of the pore or the applied voltage, we can observe a strong correlation between Δ*t* and the surface area (~0.90 in mean). Conversely, the correlation between the Δ*t* and Δ*I/I*_0_ is low (<0.75). This low correlation degree is also observed between the surface and the Δ*I/I*_0_. This could be explained by the current fluctuation during the blockade due to the DNA motion inside the pore. Interestingly, the right and the left slopes do not appear to be correlated to each other (~−0.20 in mean) nor with other parameters.

**Figure 5:**
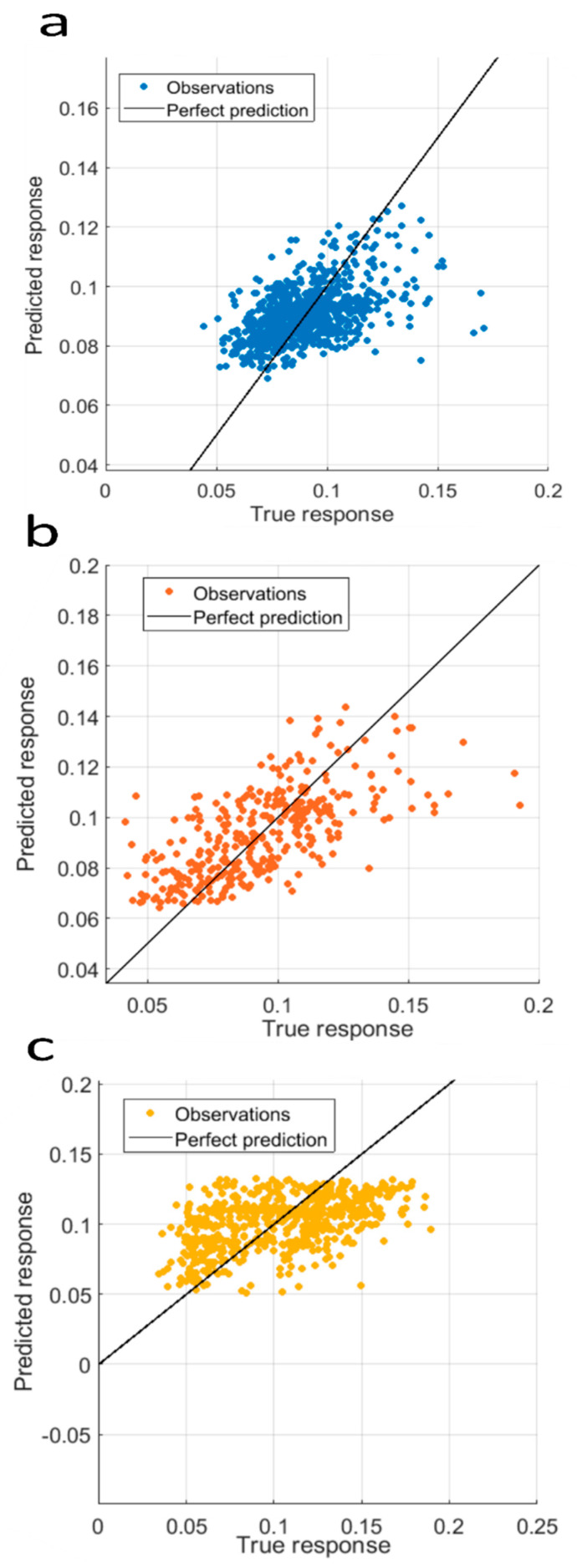
Linear regression performed with *I/I*_0_ (response variable) and Δ*t* (predictor) for (**a**) A_10_/T_10_ (R² = 0.23; n = 793), (**b**) A_40_/T_40_ (R² = 0.43; n = 332) (**c**) T_40_ (R² = 0.23; n = 634). The results were obtained using the pore *d_t_* = 3 nm, *d_b_* = 200 nm, voltage of 500 mV.

We then attempted to improve sample discrimination using machine learning algorithms. The simplest model involves establishing a linear correlation between two parameters. First, we examined whether Δ*I/I*_0_ and the Δ*t* are correlated. In Figure 5 is reported the linear regression analysis performed with Δ*I/I*_0_ as response variable and Δ*t* as predictor at a voltage of 500 mV for pore 1. We can observe a low correlation between these two parameters according to a R² about 0.25 in mean. The same analysis for the event recorded at 250 mV and with pore 2 (Figure S6) also provides a low R^2^ value (about 0.21). This is in good agreement with the heat map and confirms the non-linearity between the Δ*I/I*_0_ and the Δ*t*.

The support vector machine is a class of machine learning algorithm used to solve classification problems. The data training involves finding a way to separate the different samples by using the different parameters that characterize the events. In our case, we have defined the sample A_10_/T_10_, A_40_/T_40_ or T_40_ as the target of the algorithm. We have defined as the features the different parameters that characterize the events (Δ*I/I*_0_, Δ*t*, AUC, RS and RL). As previously mentioned, the Δ*I/I*_0_, Δ*t* are the most commonly used. Thus, we trained the algorithm with these two features and then with five features in order to demonstrate that the added parameters will help to improve the discrimination and to classify the different samples.

**Figure 6:**
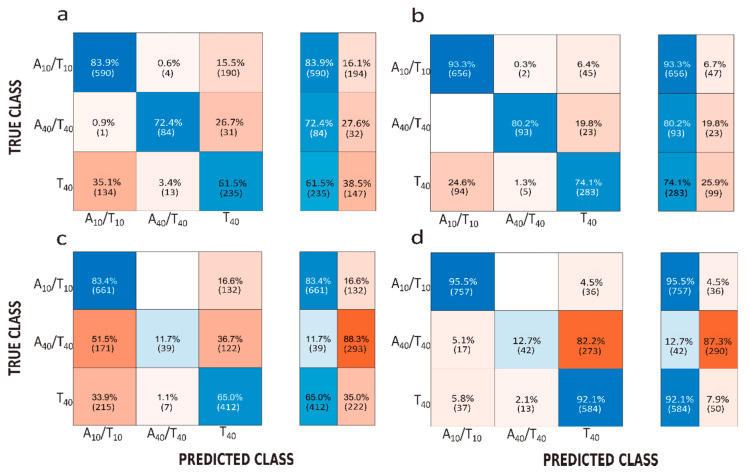
Confusion matrices representing the accuracy of classification with the support vector machine approach obtained under V = 250 mV using (**a**) 2 features (Δ*I/I*_0_, Δ*t*) and (**b**) 5 features (Δ*I/I*_0_, Δ*t*, *AUC, LS, RS*) and under 500 mV using (**c**) 2 features (Δ*I/I*_0_, Δ*t*) and (**d**) 5 features (Δ*I/I*_0_, Δ*t*, *AUC, LS, RS*). The results were obtained using pore 1 *d_t_* = 3 nm, *d_b_* = 200 nm.

In Figure 6 is reported the confusion matrix for pore 1. Using two features (*I/I*_0_ and Δ*t*), the accuracy is 72.6% and 53% for the event recorded at 250 mV and 500 mV, respectively. First, we observe that using machine learning the accuracy is better at 250 mV making this voltage more relevant to discriminate samples. Ignoring the voltage, the best predictions of event parameters were found for the A_10_/T_10_. As expected, the use of five features allows for improving the accuracy up to 82.5% and 66.76% at 250 mV and 500 mV, respectively. This improvement of the accuracy is also observed for pore 2 (Figure S7). This weak difference in precision between the two pores is likely due to the different geometries. The best results were obtained with the smaller nanopores. Using five features, the ratio of the true positive is higher for the A_10_/T_10_ and T_40_ than for the A_40_/T_40_. This is observed for all experiments except for pore 2 at 250 mV. However, this could be explained by a low number of events (n = 80).

## 4. Conclusions

In summary, three DNA samples named A_10_/T_10_, A_40_/T_40_ or T_40_ were detected using conical nanopore with a tip diameter of about 3 nm. The classical parameters used to characterize the event (*I/I*_0_ and Δ*t*) do not allow one to discriminate the samples due to a large overlap of their distributions. In addition, the linear regression analysis shows no correlation between these two parameters. Using support vector machines, the different samples were discriminated with accuracy between 50% and 72.6%. The events were then characterized by five parameters that are not correlated to each other except for Δ*t* and the surface area. The introduction of three additional parameters as features (AUC, LS, RS) in the support vector machine algorithm showed 10% improvement of accuracy, which increased to 82.5% for the smallest nanopore at 250 mV. Among the three samples, the best prediction of event parameters was found for the A_10_/T_10_.

More generally, our work was motivated to propose a solution to improve the resolution of conical track-etched nanopore. The combination of additional parameters and support vector machine algorithms was found to be a relevant solution to reach this goal. We could expect that such analysis methodology will be applied for single molecule sensing using track-etched nanopores, especially in fields where these nanopores could bring fundamental insights, such as in amyloid detection and characterization.

## Supplementary Materials

The following are available online at https://www.mdpi.com/2079-6374/10/10/140/s1. Figure S1: Distribution histograms of Δ*I/I*_0_ and Δ*t* recorded with pore 1 at 250 mV, Figure S2: Distribution histograms of Δ*I/I*_0_ and Δ*t* recorded with pore 2 at 500 mV, Figure S3: Distribution histograms of Δ*I/I*_0_ and Δ*t* recorded with pore 2 at 250 mV, Figure S4: Scatter plot representing the Δ*t* versus the Δ*I/I*_0_ recorded with the pore 2, Figure S5: Heat map representing the correlation between the variables characterizing events obtained under 250 mV. Figure S6: Linear regression performed with Δ*I/I*_0_ (response variable) and Δ*t* (predictor), Figure S7: Confusion matrix representing the accuracy of classification with the support vector machine approach obtained under V = 250 mV.
