# Supplementary material for: Machine Learning to Improve the Sensing of Biomolecules by Conical Track-Etched Nanopore
Source: Biosensors (Basel). 2020 Oct 5;10(10):140. doi: 10.3390/bios10100140 (PMC7601669; doi:10.3390/bios10100140)
Supplement: Supplementary file 1 [file biosensors-10-00140-s001.pdf]

Supplementary information: Machine learning to improve the sensing of biomolecules by conical track-etched nanopore

Nathan Meyer, Jean-Marc Janot, Mathilde Lepoitevin, Michael Smietana, Jean-Jacques Vasseur, Joan Torrent, Sebastien Balme

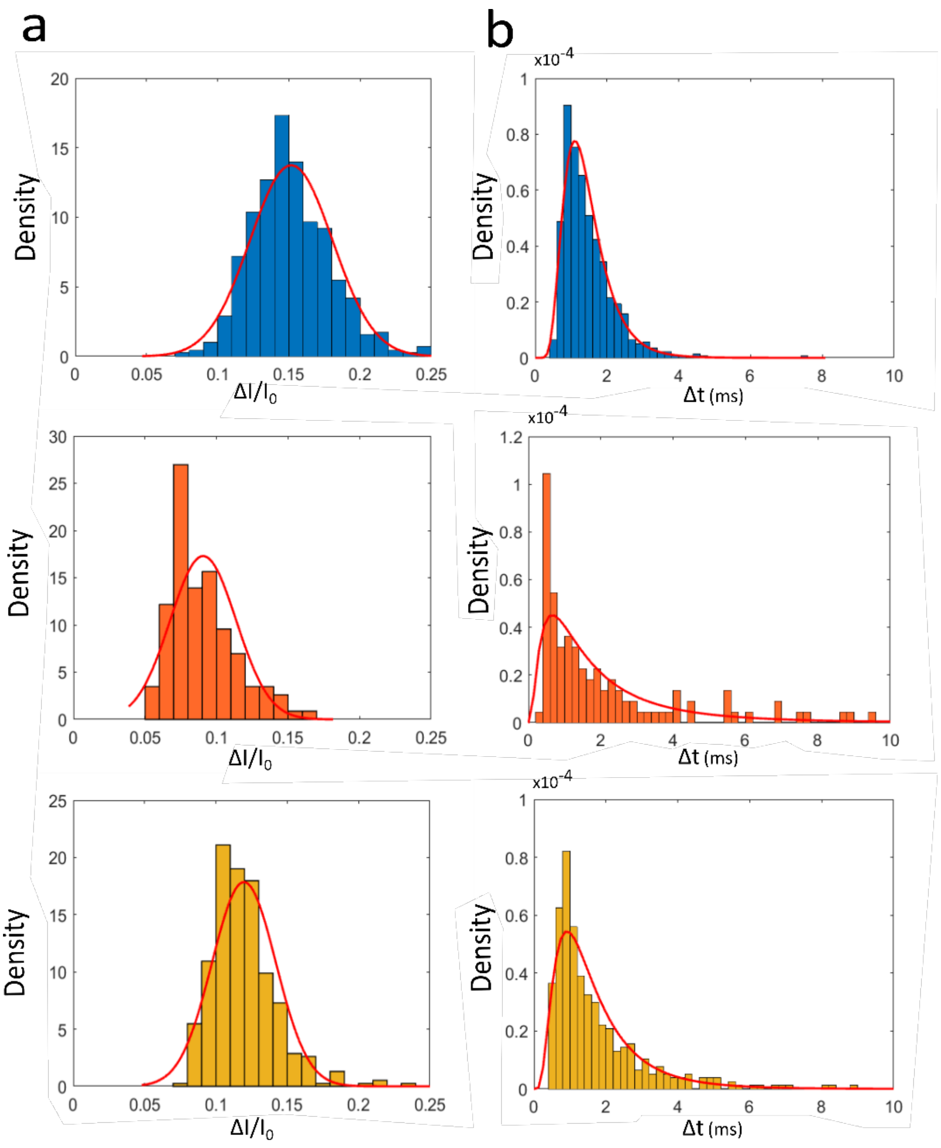

Figure S1 : Distribution histograms of a) the amplitude of the relative current blockade ($\Delta I/I_0$) and b) the dwell time (Δt) for the A_{10}/T_{10} (blue), A_{40}/T_{40} : (orange), T_{40} (yellow). The events were recorded at 250 mV with the pore 1, the number of events n= 703, 116 and 388 for A_{10}/T_{10}, A_{40}/T_{40} and T_{40} respectively). The results were obtained using the pore 1 (d_t=3 nm, d_b=200 nm). The density (d_i) is the frequency (f_i) of event relative to the sample size (n) and the bin width (w_i) $d_i = {f_i}/{nw_i}$ where w_i = 0.01 and 200 for $\Delta I/I_0$ and Δt respectively.

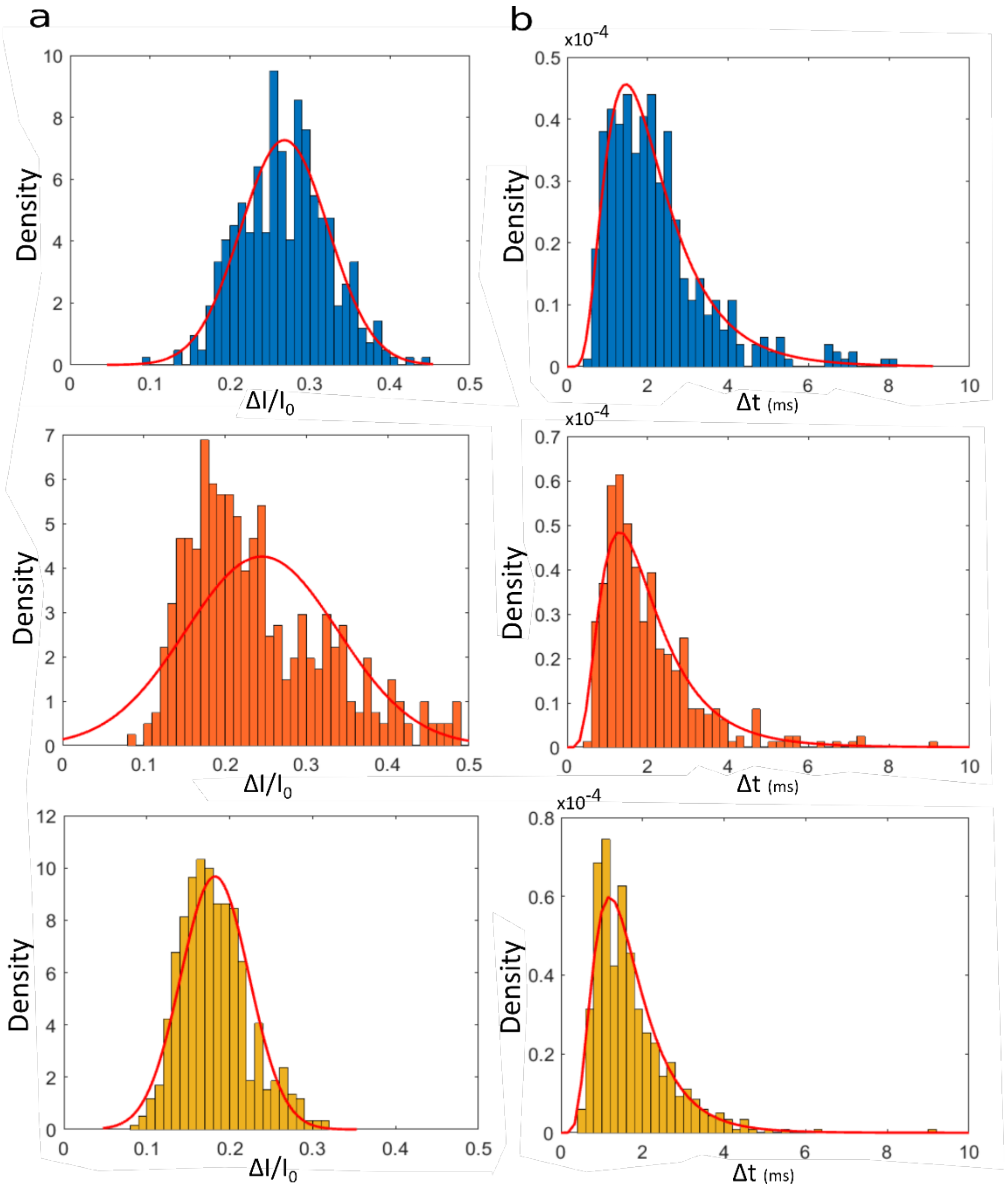

Figure S2: Distribution histograms of a) the amplitude of the relative current blockade ($\Delta I/I_0$) and b) the dwell time (Δt) for the A_{10}/T_{10} (blue), A_{40}/T_{40} : (orange), T_{40} (yellow). The events were recorded at 500 mV with the pore 2, the number of events is 421, 410 and 590 for A_{10}/T_{10}, A_{40}/T_{40} and T_{40} respectively). The results were obtained using the pore 2 (d_t=4 nm, d_b=350 nm). The density (d_i) is the frequency (f_i) of event relative to the sample size (n) and the bin width (w_i) $d_i = {}^{f_i}/_{nw_i}$ where w_i = 0.01 and 200 for $\Delta I/I_0$ and Δt respectively.

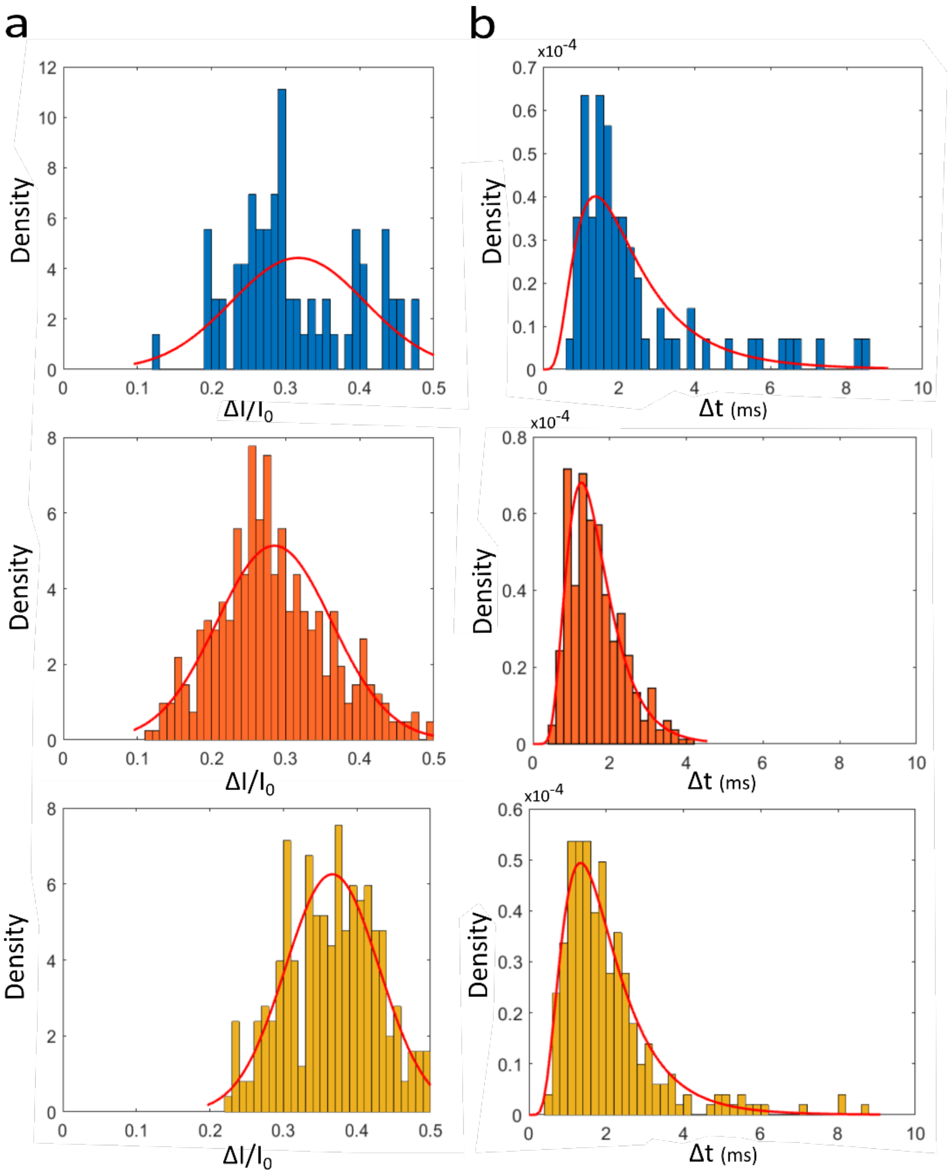

Figure S3 : Distribution histograms of a) the amplitude of the relative current blockade ($\Delta I/I_0$) and b) the dwell time (Δt) for the A_{10}/T_{10} (blue), A_{40}/T_{40} : (orange), T_{40} (yellow). The events were recorded at 250 mV with the pore 2, the number of events is 80, 415 and 253 for A_{10}/T_{10}, A_{40}/T_{40} and T_{40} respectively). The results were obtained using the pore 2 (d_t=4 nm, d_b=350 nm). The density (d_i) is the frequency (f_i) of event relative to the sample size (n) and the bin width (w_i) $d_i = {f_i}/{nw_i}$ where w_i = 0.01 and 200 for $\Delta I/I_0$ and Δt respectively.

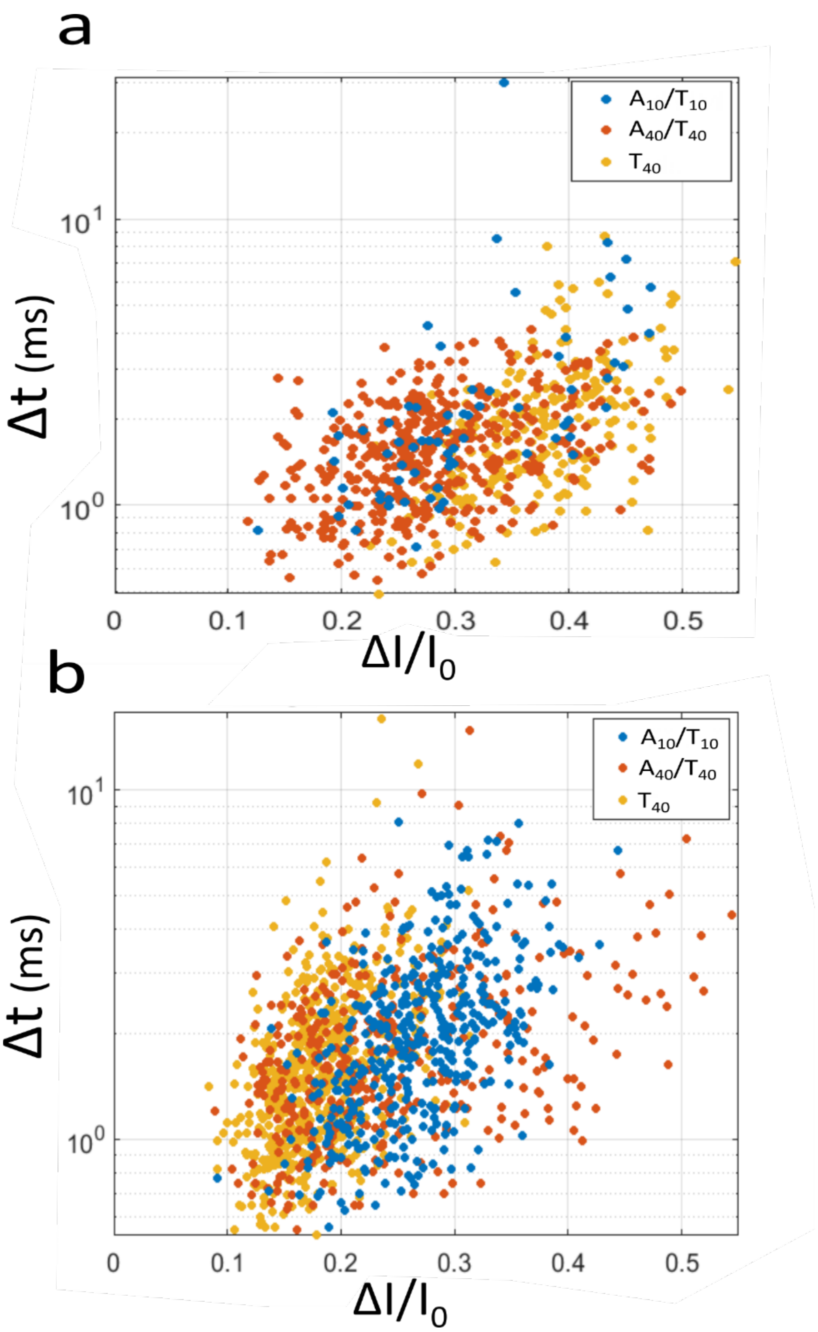

Figure S4 : Scatter plot representing the Δt versus the $\Delta I/I_0$ for the A_{10}/T_{10} (blue), A_{40}/T_{40} : (orange), T_{40} (yellow) for a voltage of (a) 250 mV and (b) 500 mV. The events were recorded with the Pore 1. The number of events recorded at 250 mV n= 80, 415 and 253 and at 500 mV n= 421, 410 and 590 for A_{10}/T_{10}, A_{40}/T_{40} and T_{40} respectively. The results were obtained using the pore 2 (d_t=4 nm, d_b=350 nm)

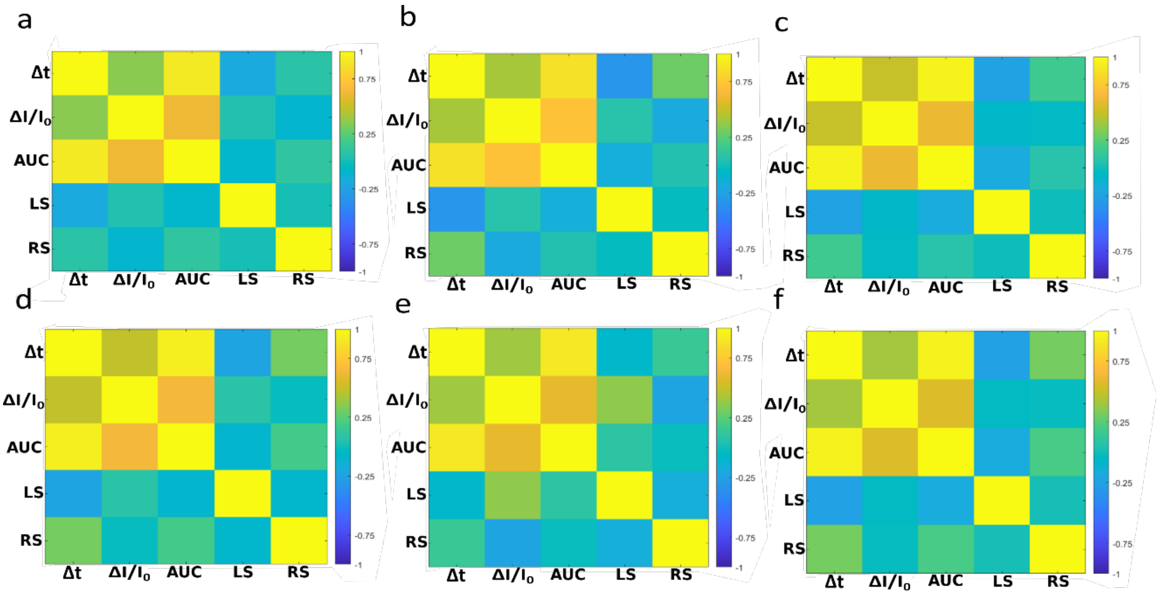

Figure S5 : Heat map representing the correlation between the variables characterizing events obtained under 250 mV for –(b) A_{10}/T_{10}, (c) A_{40}/T_{40}, (d) T_{40} and at 500 mV for (e) A_{10}/T_{10}, (f) A_{40}/T_{40}, (g) T_{40}. The results were obtained using the pore 2 (d_t=4 nm, d_b=350 nm)

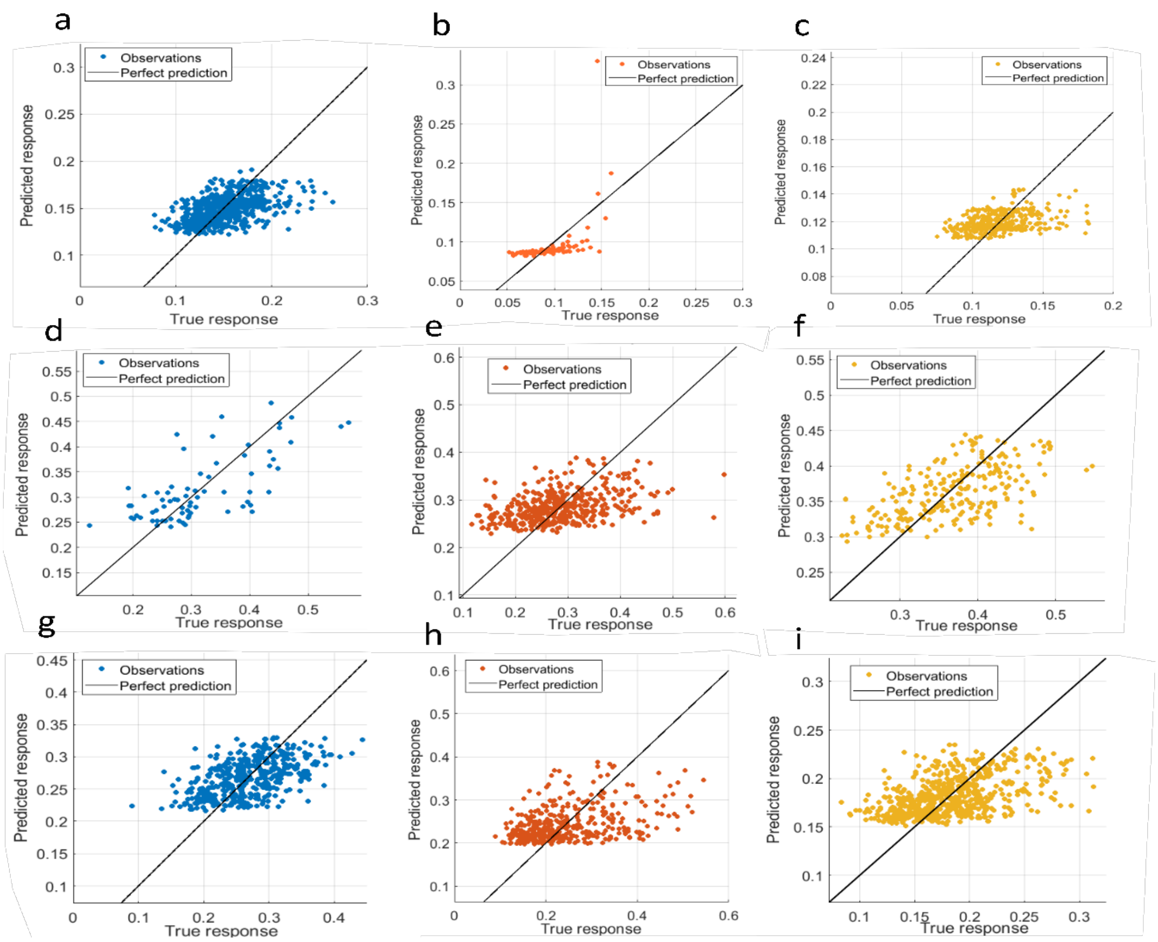

Figure S6 : Linear regression performed with $\Delta I/I_0$ (response variable) and Δt (predictor) The results show here were obtained with pore 1 (d_t=3 nm, d_b=200 nm) under V= 250 mV a) A_{10}/T_{10} (R^2 of X n = 703), b) A_{40}/T_{40} R^2= -0.12; n = 116), c) T_{40} (R^2 = 0.12; n = 382) ; pore 2 (d_t=4 nm, d_b=350 nm) under V= 250 mV d) A_{10}/T_{10} (R^2 = 0.50; n = 80), e) A_{40}/T_{40} (R^2= 0.17; n = 415), f) T_{40} (R^2 =0.33 ; n = 253) and pore 2 (d_t=4 nm, d_b=350 nm) under V= 500mV g) A_{10}/T_{10} (R^2 = 0.27; n = 421), h) A_{40}/T_{40} (R^2= 0.19; n = 4_{10}), i) T_{40} (R^2=0.23; n = 590)

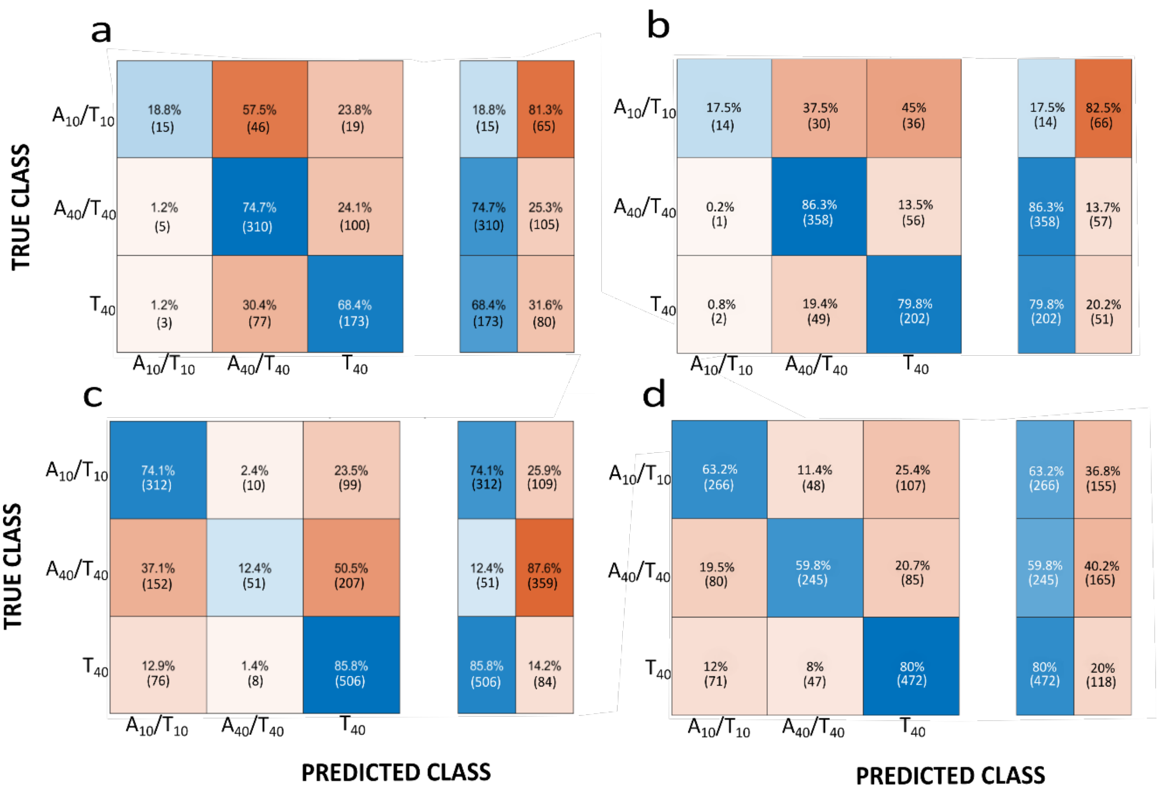

Figure S7 : Confusion matrix representing the accuracy of classification with the support vector machine approach obtained under V=250 mV using a) 2 features ($\Delta I/I_0$, Δt) and b) 5 features ($\Delta I/I_0$, Δt, *AUC, LS, RS*) and under 500 mV using c) 2 features ($\Delta I/I_0$, Δt) and d) 5 features ($\Delta I/I_0$, Δt, *AUC, LS, RS*). The results were obtained using the pore 2 (d_t=4 nm, d_b=350 nm)
